# Optimal Bladder Volume for Hypofractionated Proton Therapy in Each Localized Prostate Cancer Risk Group

**DOI:** 10.7759/cureus.48723

**Published:** 2023-11-13

**Authors:** Yuki Narita, Takahiro Kato, Yojiro Ishikawa, Kimihiro Takemasa, Hisashi Yamaguchi, Masao Murakami

**Affiliations:** 1 Department of Radiation Physics and Technology, Southern Tohoku Proton Therapy Center, Koriyama, JPN; 2 School of Health Sciences, Fukushima Medical University, Fukushima, JPN; 3 Department of Radiation Oncology, Tohoku Medical and Pharmaceutical University Hospital, Sendai, JPN; 4 Department of Radiation Oncology, Southern Tohoku Proton Therapy Center, Koriyama, JPN

**Keywords:** hypofractionated rt, radiotherapy (rt), proton therapy, bladder volume, prostate cancer

## Abstract

Background

This study aimed to determine the optimal bladder volume (BV) for hypofractionated proton therapy in prostate cancer (PC).

Materials and methods

Two hundred patients with PC were enrolled in this study and classified into low-, intermediate-, and high-risk groups. Treatment planning was performed by assuming a hypofractionated schedule of 63 Gy (relative biological effectiveness) in 21 fractions. The dose indices of the bladder (V_60_ and V_50_) were calculated and classified into four groups according to the BV. A cutoff value with a 95% confidence interval was calculated on the basis of the mean and standard deviation of the dose indices. These values were compared with the dose constraints (V_60 _< 15 % and V_50_ < 30 %).

Results

The dose indices were higher in the high-risk group than in the other risk groups. The cutoff value exceeded dose constraints in the low- and intermediate-risk groups with a BV of ≦ 149 cc. Additionally, the cutoff value exceeded the dose constraint in the high-risk group with a BV of ≦ 199 cc. In all the cases, the group with a BV of ≧ 200 cc was below the dose constraint.

Conclusions

In this study, the relationship between the dose and volume of the bladder in hypofractionated PT for PC was evaluated using a 95% CI to determine the optimal BV. The BV should be changed for each risk group, and a larger BV is required for a high-risk group than for other risk groups.

## Introduction

In radiotherapy (RT) for prostate cancer (PC), it is desirable to maintain target coverage while keeping the dose of organs at risk (OARs), such as the rectum, bladder, urethra, and femoral head within a tolerance level. With advances in RT techniques, intensity-modulated RT (IMRT) and volumetric-modulated arc therapy (VMAT) can deliver high doses to the target and minimize the dose to the OARs [1-4]. Proton therapy (PT) has attracted attention because of the distinct physical properties of proton beams, which can deposit high doses on the target without an exit dose away from the target, further reducing the dose to the OARs [5,6]. In recent years, hypofractionated RT for PC has become the standard of care when the target is limited to the prostate and seminal vesicles (SVs). Several reports have shown that moderate or ultra-hypofractionated RT (high dose per day) provides good biochemical control that is comparable to conventional fractionated RT [7-11].

In RT for PC, the management of the rectum and bladder is important for effective and safe treatment. The rectum can maintain reproducibility through defecation, suction of gas in the rectum, and administration of laxatives. Many institutions instruct patients to keep their bladders full for certain periods. Because of differences in this preparation among institutions and in bladder filling among individuals, the bladder volume (BV) is not uniform, resulting in a dose constraint that cannot be met during treatment planning. During computed tomography (CT) for treatment planning, the appropriateness of the BV must be determined; however, there is currently no consensus on the optimal BV in RT for PC. Furthermore, the target delineation of PC differs for each risk group [12,13]. Because the bladder is located above the prostate and SVs, the BV exposed to radiation changes, depending on whether SVs are included in addition to the prostate as the clinical target volume (CTV); the required BV is expected to change accordingly. Therefore, we investigated the optimal BV for hypofractionated PT in each localized PC risk group.

## Materials and methods

Imaging and Planning Procedure

**Figure 1:**
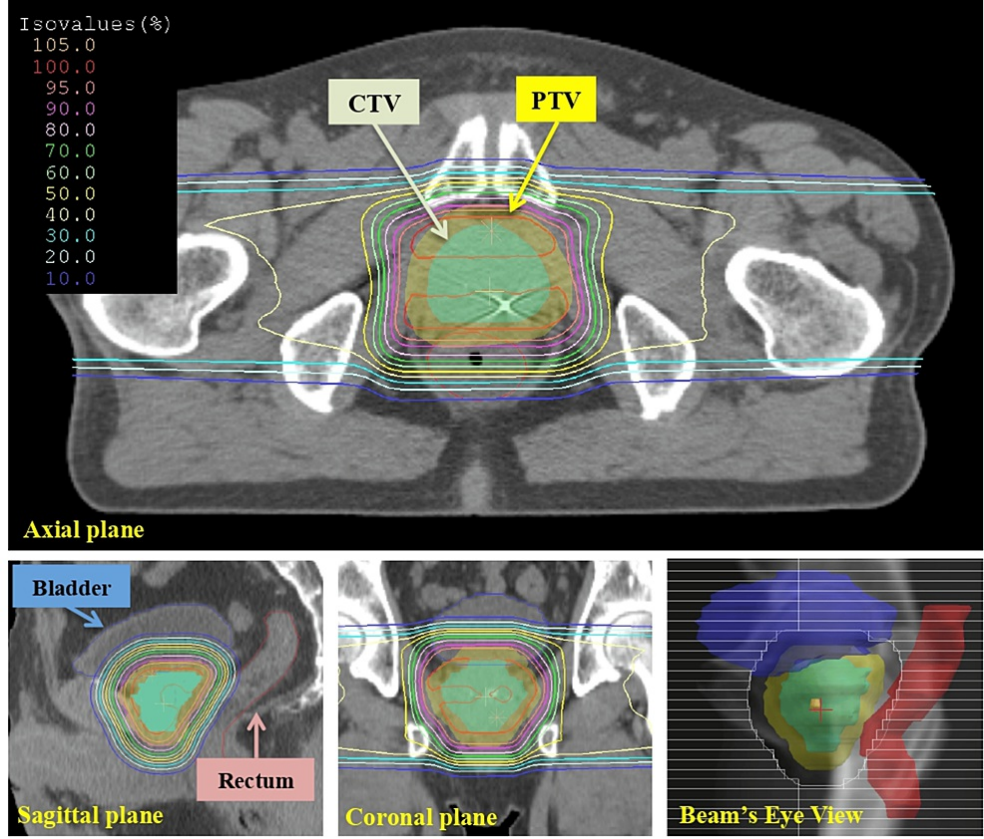
Dose distribution of proton therapy CTV, Clinical Target Volume PTV, Planning Target Volume

Between September 2019 and September 2022, 200 patients who underwent passive scattering PT (PSPT) at our institution for localized PC without distant metastasis were enrolled in this study. Hitachi proton-type particle therapy system was used for PT. This study was approved by our institutional review board. Gold fiducial markers (Gold Anchor, Naslund Medical Aktiebolag, Huddinge, Sweden) were implanted in the prostate to accurately irradiate the target, and a hydrogel spacer (SpaceOAR, Boston Scientific, Marlborough, United States of America) was inserted between the prostate and rectum to reduce the dose to the rectum. The patients were placed in the supine position, and their lower legs were fixed with a vacuum cushion to reproduce the femoral head. Thirty minutes before CT imaging for treatment planning and daily irradiation, all patients were instructed to void and drink 200 cc of water. The Aquilion Large Bore (Canon Medical Systems, Tochigi, Japan) CT scanner was used, and images were obtained with a 2-mm slice thickness. Additionally, magnetic resonance images (MRI) with a 4-mm slice thickness were obtained using the Signa HDx (General Electric Healthcare, Milwaukee, Wisconsin, United States of America) scanner and registered to the CT images to more accurately delineate the target volume and OARs. The images were imported into the XiO−M treatment planning system (Hitachi, Tokyo, Japan), and a physician manually contoured the target volume and OARs for all the patients. The rectum, bladder, femoral head (left and right), and small and large bowels were contoured as the OARs. Based on the National Comprehensive Cancer Network guidelines for PC [13], 37, 85, and 78 patients were classified into low-, intermediate-, and high-risk groups, respectively. CTV was defined as the prostate and SVs, with only the prostate at low risk. At intermediate risk, the base of the SVs was included in the CTV. At high risk, depending on the clinical tumor (T) stage, one-third of the SVs (< T3), two-thirds of the SVs (T3a), or the entire SVs (T3b) were included in the CTV. The planning target volume (PTV) was defined as the CTV with a 7-mm safety margin (the posterior margin was 6 mm to decrease the risk of rectal toxicity). As key parameters for PSPT planning, the distal, proximal, and lateral margins, and compensator smear were calculated using the formulas proposed by Moyers et al. [14]. The penumbra (7 mm) and setup (5 mm), range (3 mm), and Hounsfield unit uncertainties (3.5 %) were considered in the margin calculations and used in the CTV. The PT plans consisted of laterally opposed fields with 210-MeV proton beams (Figure 1). The wobbler and ridge filter method, which is a passive scattering method, was used for the field design. A hypofractionated schedule was assumed for all the patients. As this was a simulation study, the total prescription dose was 63.0 Gy relative biological effectiveness (RBE) in 21 fractions, regardless of risk classification. The RBE collection factor was 1.1. All plans were normalized so that 100% of the PTV received 95% of the prescribed dose. The maximum dose of the PTV was restricted to 110% of the prescribed dose. The dose to each OAR was reduced on the basis of the dose constraint used in treatment planning at our institution. The rectum was limited to the volume receiving 60, 50, and 30 Gy (RBE) (V_60_ < 10, V_50_ < 20, and V_30_ < 30%, respectively). The dose administered to the femoral head was limited to 45 Gy (RBE). The small and large bowels were limited to the volume receiving 50 Gy (RBE) (V_50_ < 0.5 cc); however, no dose constraints were applied to the bladder in this study.

Analysis of Bladder Dose and Statistics

The bladder dose (BD) was calculated for each risk group. It was assessed using the BV receiving 60 and 50 Gy (RBE) (V_60_ and V_50_, respectively). The differences in the doses of V_60_ and V_50_ were compared for each risk group. The BD for each risk group was further divided into the following four subgroups according to the BV: < 100 cc (group 1), 100−149 cc (group 2), 150−199 cc (group 3), and ≥ 200 cc (group 4). The upper limit of the 95% confidence interval (CI) was calculated as the cutoff value on the basis of the mean and standard deviation (SD) of the BD in each group (mean + 1.96 SD). This cutoff value was compared with the dose constraint of the bladder (V_60_ < 15 % and V_50_ < 30 %) used at our institution to determine the minimum tolerance of the BV. The dose constraint values of V_60_ and V_50_ were based on those in a clinical trial of hypofractionated RT for PC [15,16]. For descriptive statistics, the Wilcoxon matched-pairs nonparametric test was used to analyze the differences in the mean V_60_ and V_50 _for each group. Statistical p-values of < 0.05 were considered significant.

## Results

Patient characteristics are shown in Table 1. 

**Table 1:** Patient characteristics NCCN, National comprehensive cancer network; CTV, Clinical tumor volume

Characteristic	Value
Total	200
Age at diagnosis (years) [median (range)]	
Median	71 (46-87)
PSA (ng/dL) [median (range)]	
Median	7.4 (1.4-77.8)
Gleason score	
≤ 6	57
7	82
8 and above	61
Tumor stage	
T1-2a	121
T2b	15
T2c	32
T3a	21
T3b	10
T4	1
NCCN risk group	
Low	37
Intermediate	85
High	78
CTV volume (cc) [median (range)]	
Low (n = 37)	28.8 (13.7 - 79.7)
Intermediate (n = 85)	33.2 (23.8 - 124.0)
High (n = 78)	38.9 (19.8 - 98.8)
Bladder volume (cc) [median (range)]	
Low (n = 37)	138.0 (60.3-390.5)
Intermediate (n = 85)	119.9 (61.8-404.0)
High (n = 78)	124.9 (48.4-433.4)

**Figure 2:**
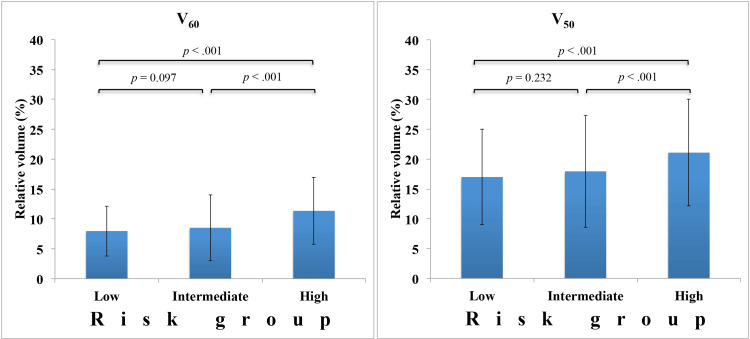
Comparison of mean bladder doses by risk group The left and right figures show the mean bladder dose for V_60_ and V_50_, respectively. The error bars indicate the standard deviation in each risk group. *P *values between each risk group are indicated above. Significant at *p* < 0.05.

Based on the National Comprehensive Cancer Network (NCCN) guidelines for PC [13], 37, 85, and 78 patients were classified into low-, intermediate-, and high-risk groups, respectively. Table 2 shows the dose-volume data for the bladders of the 200 patients evaluated by risk groups. The median BV for all the patients was 129.2 cc (range: 48.4−433.4) and did not differ by risk group. In the evaluations of V_60_ and V_50_, each dose tended to increase with increasing risk. Figure 2 shows the comparison between V_60_ and V_50_ for each risk group. There was no statistically significant difference between the low- and intermediate-risk groups (*p* = 0.097 at V_60_ and *p* = 0.232 at V_50_, respectively); however, a statistically significant difference was observed between the high-risk and the other groups (*p* < 0.001). In all cases, the mean values of each group did not exceed the dose constraints.

**Table 2:** Dose-volume data for the bladder n, Number of patients; V_60_ and V_50_, Volume of bladder receiving 60 and 50 Gy (RBE), respectively; RBE, Relative biological effectiveness; SD, Standard deviation

Value	
Bladder volume (cc) [median (range) ]	
Total (n = 200)	129.2 (48.4-433.4)
Low risk (n = 37)	138.0 (60.3-390.5)
Intermediate risk (n = 85)	119.9 (61.8-404.0)
High risk (n = 78)	124.9 (48.4-433.4)
Bladder dose_V60 (%) (mean ± SD)	
Total (n = 200)	9.4 ± 5.3
Low risk (n = 37)	8.0 ± 4.2
Intermediate risk (n = 85)	8.5 ± 5.5
High risk (n = 78)	11.3 ± 5.6
Bladder dose_V50 (%) (mean ± SD)	
Total (n = 200)	18.8 ± 8.9
Low risk (n = 37)	17.0 ± 8.0
Intermediate risk (n = 85)	17.9 ± 9.4
High risk (n = 78)	21.1 ± 8.9

Table 3 shows the cutoff value (mean + 1.96SD) calculated from the upper limit of the 95 % CI in V_60_ and V_50_. In all the cases, the dose constraint was exceeded (groups 1, 2, and 3), with a BV of ≤ 199 cc. In the evaluation of each risk group, the dose constraint was exceeded in the low- and intermediate-risk groups with a BV of ≤ 149 cc (groups 1 and 2) and in the high-risk group with a BV of ≤ 199 cc (groups 1, 2, and 3). In all the cases, the group with a BV of ≧ 200 cc was below the dose constraint.

**Table 3:** Cutoff values (mean + 1.96 SD) of V60 and V50 for the bladder V_60_ and V_50_, Volume of bladder receiving 60 and 50 Gy (RBE), respectively; SD, Standard deviation; n, Number of patients

Total (n=200)				
Bladder volume	n		V60 (%)	V50 (%)
< 100 cc (group 1)	46		23.1	41.6
100-149 cc (group 2)	76		20.7	36.5
150-199 cc (group 3)	40		16.6	30.1
≥ 200 cc (group 4)	38		11.9	19.5
Low risk group (n=37)				
Bladder volume	n		V_60_ (%)	V_50_ (%)
< 100 cc (group 1)	8		25.1	46.3
100-149 cc (group 2)	15		16.7	30.3
150-199 cc (group 3)	8		13.1	26.9
≥ 200 cc (group 4)	6		13.3	19.5
Middle risk group (n=85)				
Bladder volume	n		V_60_ (%)	V_50_ (%)
< 100 cc (group 1)	16		17.6	36.7
100-149 cc (group 2)	34		17.8	34.9
150-199 cc (group 3)	16		13.9	24.5
≥200 cc (group 4)	19		10.5	19.3
High risk group (n=78)				
Bladder volume	n		V_60_ (%)	V_50_ (%)
< 100 cc (group 1)	22		24.8	42.3
100-149 cc (group 2)	27		24.3	40.3
150-199 cc (group 3)	16		18.2	33.2
≥ 200 cc (group 4)	16		12.8	19.3

## Discussion

In this study, the optimal BV for PC was evaluated through a retrospective analysis of 200 patients treated with hypofractionated PT. The mean and SD of the V_60_ and V_50_ for each risk group were compared. Although the high-risk group received significantly higher doses than the others, the mean value for each group did not exceed the dose constraints. Furthermore, in this study, the optimal BV, which is an interval estimation method, was evaluated using a 95% CI. Consequently, the dose constraint was exceeded in the subgroups (groups 1,2) of the low- and intermediate-risk groups with a BV of ≤ 149 cc and the subgroups (groups 1−3) of the high-risk group with a BV of ≤ 199 cc. Therefore, the optimal BV was ≥ 150 cc in the low- and intermediate-risk groups, and ≥ 200 cc in the high-risk group in the treatment planning for hypofractionated PT for PC. This is presumably because the range of SVs included in the CTV differs for each risk factor; the BV exposed to radiation tends to be larger in high-risk groups. Thus, different BVs are required, depending on the risk group for PC. However, to date, the optimal BV for treatment planning is unclear. Most facilities instruct patients to fill their bladders by voiding and drinking water prior to imaging and treatment. Although this method was uniformly applied in all patients, the BV obtained differed depending on individual differences. Therefore, without setting the appropriate criteria for the BV, BD may be inappropriate at the time of treatment planning. We believe our study will provide useful information to many institutions in deciding whether the BV meets the criteria at the time of CT imaging for treatment planning, or whether preparations such as urination and drinking water should be reviewed. There are several reports on the optimal BV, with Fujioka et al. reporting that a BV of 100−250 cc is ideal, as evaluated using VMAT [17], and Nakamura et al. reporting that a BV of ≥150 did not improve with the tomotherapy treatment plan [18]. In these reports, optimal BV was determined by evaluating V_70_ for a prescribed dose of 74.0−76.0 Gy in 37−38 fractions, and in this study, it was also evaluated using dose indices of V_60_ and V_50_ in the same high-dose range (≥ 80−90% of the prescription dose). A previous study comparing the dose distribution of PT with those of VMAT and tomotherapy reported that PT significantly reduced BD in the low- and medium-dose ranges; however, no difference was observed in the high-dose range [19, 20]. Therefore, we believe that the BV obtained in our study was similar to that evaluated at other institutions. However, we presented the optimal BV in hypofractionated PT planning and evidence that the value should be varied according to the risk group. PSPT was used for evaluation in this study; however, in recent years, the number of facilities that treat PC using the pencil beam scanning (PBS) technique has increased. A comparison of the dose distributions of PSPT and PBS showed that although there was no significant difference in the maximum BD, compared with PSPT, PBS significantly reduced the low- to medium-dose range [20]. Therefore, as PBS and PSPT presented similar trends in the high-dose range, we believe that the results of this study are also useful for PBS.

When the 200 patients evaluated in this study were observed individually, there were some cases wherein the dose constraints were met even if the BV was less than the criteria. Even if the BV is identical, the irradiation range of the bladder varies substantially as the individual bladder shape is different when urine accumulates. However, if the BV is too small, the risk to the small bowel, including the irradiation field, increases. Therefore, the BV should ideally be as large as possible. In clinical practice, some cases find holding urine for a prolonged time difficult because of older age, prostatic hyperplasia, and other factors. Several studies have reported that patients with a large initial BV tend to have large changes in the BV during treatment [21]. Therefore, even if the BV is exceeded on CT imaging, reproducing the volume during treatment may be difficult. Additionally, many studies have shown that inconsistent BV variations are a risk factor for changing prostate and SV positions and increasing doses to OARs [22-24], and minimizing this interfractional variation is important. A limitation of this study is that this variation was not considered in setting the optimal BV. Bladder volume must be set to a reproducible value for each patient, but this value could not be fully evaluated in this study. Recently, an increasing number of institutions have introduced ultrasound image diagnosis devices to confirm whether the required BV has been achieved during irradiation or imaging [25-27]. However, when using such devices, there are problems such as measurement errors between observers. Whether introducing them is possible must be considered at each institution.

If it is not possible to secure a sufficient BV and the dose constraint cannot be met, the treatment plan must be reviewed. Particularly, a reduction in the PTV margin must be considered. At our institution, when hypofractionated PT is performed, gold fiducial markers are implanted into the prostate, and the position of the prostate is corrected by matching the marker before irradiation. The correction of position using markers can improve prostate localization; therefore, the PTV margin is expected to be reduced [28,29]. Consequently, a reduction in the dose irradiated to the bladder can be expected. However, the positional displacement of the SV may not be accurately corrected. A PTV margin reduction may increase the risk of geographic misses and result in reduced local control and survival rates. Therefore, these reductions must be appropriate and should only be performed when appropriate measures are taken to ensure that the PTV margins can be safely reduced.

## Conclusions

In this study, the relationship between the BV and BD in hypofractionated PT for PC was evaluated using a 95 % CI to determine the optimal BV. The BV should be changed for each risk group, and a larger BV is required for high-risk groups than for other risk groups.

## References

[1] Sale C, Moloney P (2011). Dose comparisons for conformal, IMRT and VMAT prostate plans. J Med Imaging Radiat Oncol.

[2] Fenoglietto P, Laliberte B, Allaw A (2008). Persistently better treatment planning results of intensity-modulated (IMRT) over conformal radiotherapy (3D-CRT) in prostate cancer patients with significant variation of clinical target volume and/or organs-at-risk. Radiother Oncol.

[3] Wolff D, Stieler F, Welzel G (2009). Volumetric modulated arc therapy (VMAT) vs. serial tomotherapy, step-and-shoot IMRT and 3D-conformal RT for treatment of prostate cancer. Radiother Oncol.

[4] Tsai CL, Wu JK, Chao HL, Tsai YC, Cheng JC (2011). Treatment and dosimetric advantages between VMAT, IMRT, and helical tomotherapy in prostate cancer. Med Dosim.

[5] Vargas C, Fryer A, Mahajan C (2008). Dose−volume comparison of proton therapy and intensity-modulated radiotherapy for prostate cancer. Int J Radiat Oncol Biol Phys.

[6] Dowdell SJ, Metcalfe PE, Morales JE, Jackson M, Rosenfeld AB (2008). A comparison of proton therapy and IMRT treatment plans for prostate radiotherapy. Australas Phys Eng Sci Med.

[7] Benjamin LC, Tree AC, Dearnaley DP (2017). The role of hypofractionated radiotherapy in prostate cancer. Curr Oncol Rep.

[8] Dearnaley D, Syndikus I, Sumo G (2012). Conventional versus hypofractionated high-dose intensity-modulated radiotherapy for prostate cancer: preliminary safety results from the CHHiP randomised controlled trial. Lancet Oncol.

[9] Koontz BF, Bossi A, Cozzarini C, Wiegel T, D'Amico A (2015). A systematic review of hypofractionation for primary management of prostate cancer. Eur Urol.

[10] Quinn TJ, Hamstra D (2019). Hypofractionation in prostate cancer using proton beam. Int J Radiat Oncol Biol Phys.

[11] Di Franco R, Borzillo V, Ravo V (2017). Rectal/urinary toxicity after hypofractionated vs. conventional radiotherapy in high risk prostate cancer: systematic review and meta analysis. Eur Rev Med Pharmacol Sci.

[12] D'Amico AV, Whittington R, Malkowicz SB (1998). Biochemical outcome after radical prostatectomy, external beam radiation therapy, or interstitial radiation therapy for clinically localized prostate cancer. JAMA.

[13] Mohler JL, Antonarakis ES, Armstrong AJ (2019). Prostate cancer, version 2.2019, NCCN clinical practice guidelines in oncology. J Natl Compr Canc Netw.

[14] Moyers MF, Miller DW, Bush DA, Slater JD (2011). Methodologies and tools for proton beam design for lung tumors. Int J Radiat Oncol Biol Phys.

[15] Ha B, Cho KH, Lee KH (2019). Long-term results of a phase II study of hypofractionated proton therapy for prostate cancer: moderate versus extreme hypofractionation. Radiat Oncol.

[16] Kim YJ, Cho KH, Pyo HR (2013). A phase II study of hypofractionated proton therapy for prostate cancer. Acta Oncol.

[17] Fujioka C, Ishii K, Yamanaga T (2016). Optimal bladder volume at treatment planning for prostate cancer patients receiving volumetric modulated arc therapy. Pract Radiat Oncol.

[18] Nakamura N, Shikama N, Takahashi O, Sekiguchi K, Hama Y, Akahane K, Nakagawa K (2012). The relationship between the bladder volume and optimal treatment planning in definitive radiotherapy for localized prostate cancer. Acta Oncol.

[19] Schwarz M, Pierelli A, Fiorino C (2011). Helical tomotherapy and intensity modulated proton therapy in the treatment of early stage prostate cancer: a treatment planning comparison. Radiother Oncol.

[20] Kase Y, Yamashita H, Fuji H, Yamamoto Y, Pu Y, Tsukishima C, Murayama S (2012). A treatment planning comparison of passive-scattering and intensity-modulated proton therapy for typical tumor sites. J Radiat Res.

[21] Lebesque JV, Bruce AM, Kroes AP, Touw A, Shouman RT, Herk MV (1995). Variation in volumes, dose−volume histograms, and estimated normal tissue complication probabilities of rectum and bladder during conformal radiotherapy of T3 prostate cancer. Int J Radiat Oncol Biol Phys.

[22] Pinkawa M, Asadpour B, Gagel B, Piroth MD, Holy R, Eble MJ (2006). Prostate position variability and dose−volume histograms in radiotherapy for prostate cancer with full and empty bladder. Int J Radiat Oncol Biol Phys.

[23] Mak D, Gill S, Paul R (2012). Seminal vesicle interfraction displacement and margins in image guided radiotherapy for prostate cancer. Radiat Oncol.

[24] Moiseenko V, Liu M, Kristensen S, Gelowitz G, Berthelet E (2006). Effect of bladder filling on doses to prostate and organs at risk: a treatment planning study. J Appl Clin Med Phys.

[25] Kuo DY, Hsu CY, Wang WC, Chen HH, Shueng PW (2021). BladderScan feedback method in predicting bladder filling for prostate radiotherapy: a prospective study. Technol Cancer Res Treat.

[26] Cramp L, Connors V, Wood M, Westhuyzen J, McKay M, Greenham S (2016). Use of a prospective cohort study in the development of a bladder scanning protocol to assist in bladder filling consistency for prostate cancer patients receiving radiation therapy. J Med Radiat Sci.

[27] Liang CC, Wei TY, Chang SD, Hsieh CC (2009). Bladder volume determination: two−dimensional versus three−dimensional transvaginal ultrasound. Taiwan J Obstet Gynecol.

[28] Su Z, Henderson R, Nichols R, Bryant C, Hoppe B, Mendenhall W, Mendenhall N (2021). A comparative study of prostate PTV margins for patients using hydrogel spacer or rectal balloon in proton therapy. Phys Med.

[29] Su Z, Li Z, Henderson R (2019). PTV margin analysis for prostate patients treated with initial pelvic nodal IMRT and prostate proton boost. Phys Med Biol.

